# Differential impact of tumor suppressor gene (*TP53, PTEN, RB1*) alterations and treatment outcomes in metastatic, hormone-sensitive prostate cancer

**DOI:** 10.1038/s41391-021-00430-4

**Published:** 2021-07-22

**Authors:** Miguel Gonzalez Velez, Heidi E. Kosiorek, Jan B. Egan, Andrea L. McNatty, Irbaz B. Riaz, Steven R. Hwang, Glenn A. Stewart, Thai H. Ho, Cassandra N. Moore, Parminder Singh, Renee K. Sharpsten, Brian A. Costello, Alan H. Bryce

**Affiliations:** 1 Division of Hematology and Medical Oncology, Phoenix, AZ USA; 2 Department of Quantitative Health Sciences, Scottsdale, AZ USA; 3 Center for Individualized Medicine, Mayo Clinic, Phoenix, AZ USA; 4 Division of Pharmacy, Scottsdale, AZ USA; 5 Department of Internal Medicine, Rochester, MN USA; 6 Division of Medical Oncology, Mayo Clinic, Rochester, MN USA

**Keywords:** Cancer genetics, Cancer therapy

## Abstract

**Background:**

Altered tumor suppressor genes (TSG-alt) in prostate cancer are associated with worse outcomes. The prognostic value of TSG-alt in metastatic, hormone-sensitive prostate cancer (M1-HSPC) is unknown. We evaluated the effects of TSG-alt on outcomes in M1-HSPC and their prognostic impact by first-line treatment.

**Methods:**

We retrospectively identified patients with M1-HSPC at our institution treated with first-line androgen deprivation therapy plus docetaxel (ADT + D) or abiraterone acetate (ADT + A). TSG-alt was defined as any alteration in one or more TSG. The main outcomes were Kaplan–Meier-estimated progression-free survival (PFS) and overall survival, analyzed with the log-rank test. Clinical characteristics were compared with the *χ*^2^ test and Kruskal–Wallis rank sum test. Cox regression was used for univariate and multivariable analyses.

**Results:**

We identified 97 patients with M1-HSPC: 48 (49%) with ADT + A and 49 (51%) with ADT + D. Of 96 patients with data available, 33 (34%) had 1 TSG-alt, 16 (17%) had 2 TSG-alt, and 2 (2%) had 3 TSG-alt. The most common alterations were in *TP53* (36%) and *PTEN* (31%); 6% had *RB1* alterations. Median PFS was 13.1 (95% CI, 10.3–26.0) months for patients with normal TSGs (TSG-normal) vs. 7.8 (95% CI, 5.8–10.5) months for TSG-alt (*P* = 0.005). Median PFS was lower for patients with TSG-alt vs TSG-normal for those with ADT + A (TSG-alt: 8.0 [95% CI, 5.8–13.8] months vs. TSG-normal: 23.2 [95% CI, 13.1–not estimated] months), but not with ADT + D (TSG-alt: 7.8 [95% CI, 5.7–12.9] months vs. TSG-normal: 9.5 [95% CI, 4.8–24.7] months). On multivariable analysis, only TSG-alt predicted worse PFS (hazard ratio, 2.37; 95% CI, 1.42–3.96; *P* < 0.001).

**Conclusions:**

The presence of TSG-alt outperforms clinical criteria for predicting early progression during first-line treatment of M1-HSPC. ADT + A was less effective in patients with than without TSG-alt. Confirmation of these findings may establish the need for inclusion of molecular stratification in treatment algorithms.

## Introduction

With the advent of comprehensive genomic profiling, many studies have shown the effects of tumor suppressor genes (TSGs) in advanced metastatic prostate cancer [1, 2]. Preclinical and clinical data indicate that cooperative functional losses of TSGs such as *TP53*, *PTEN*, and *RB1* are associated with worse clinical outcomes in castration-resistant prostate cancer [2–7].

Treatment decisions for newly diagnosed metastatic, hormone-sensitive prostate cancer (M1-HSPC) are usually made on the basis of clinical phenotypes, toxicity, convenience, and cost [8, 9]. Since 2015, 4 agents (docetaxel, abiraterone acetate, enzalutamide, and apalutamide) have shown improved survival for patients with M1-HSPC [10–15]. In the case of docetaxel, the CHAARTED trial (Androgen Ablation Therapy With or Without Chemotherapy in Treating Patients With Metastatic Prostate Cancer) suggested that the benefit is limited to patients with high-volume disease, and all studies showed inferior outcomes in patients with de novo metastatic disease [10, 16, 17]. However, the choice of first-line treatment is still unclear, as is any possible role for introducing molecular characteristics into management decisions.

TSG alterations (TSG-alt) are known to influence the efficacy of cytotoxic chemotherapy and androgen deprivation therapy (ADT). The genomic underpinnings of clinical phenotypes in M1-HSPC are increasingly well defined, and some TSG-alt are known to affect the aggressiveness of prostate cancer. For example, *TP53* loss is known to confer a poor response to ADT [1], *PTEN* loss is a prognostic marker for decreased response to hormonal therapy without affecting docetaxel efficacy [18, 19], and *RB1* loss is usually associated with worse outcomes with ADT [6, 20].

In the current study, we aimed to evaluate the prognostic value of TSG-alt of *TP53*, *PTEN*, and *RB1* in patients with M1-HSPC receiving first-line treatment with abiraterone acetate or docetaxel. Our findings suggest that TSG status could be used in the development of novel treatment algorithms.

## Methods

This study was approved by the Mayo Clinic Institutional Review Board. For this retrospective analysis, we searched for the records of patients treated with first-line ADT plus abiraterone acetate (ADT + A) or ADT plus docetaxel (ADT + D) for M1-HSPC between November 1, 2015, and December 23, 2018, at Mayo Clinic in Arizona. We identified consecutive patients with a diagnosis of castration-sensitive prostate cancer who also had baseline genomic testing or tissue available for retrospective analysis. Clinical characteristics, first-line treatment, and pathogenic TSG-alt were recorded.

Our study cohort included patients with either de novo metastatic disease or metastatic relapse after prior local therapy. Only patient outcomes for first-line treatment for the M1-HSPC setting were included. All patients had archival formalin-fixed, paraffin-embedded tumor tissue from a prostate biopsy and/or prostatectomy or other diagnostic biopsies obtained before the initiation of treatment. Sequencing of tumor tissue was performed using Clinical Laboratory Improvement Amendments of 1988–approved commercially available platforms—Tempus (58% of samples), Foundation Medicine (Roche; 37% of samples), and Caris Life Sciences (5% of samples)—for genes including *TP53*, *PTEN*, and *RB1*. *TSG-alt* was defined as any pathogenic alteration in one or more TSG (*TP53*, *PTEN*, or *RB1*). The definition of TSG-alt was determined according to the testing platform, and allelic status was not examined (Supplementary Table 1). Progression-free survival (PFS) was defined as the time from the start of therapy for M1-HSPC to identification of clinical or radiographic progression defined by the Prostate Cancer Working Group criteria [21]. Radiographic progression, not prostate-specific antigen (PSA) progression, was used to guide therapy, following institutional practice patterns. Overall survival (OS) was defined as the time from start of therapy until death of any cause. Patients who did not have progression of disease or death were considered censored at the date of last follow-up.

PFS and OS were compared between patients with TSG-alt and without gene alterations, *TSG-normal*. Descriptive statistics were used to compare baseline characteristics between groups according to TSG status and treatment regimen: the *χ*^2^ test for categorical data, and the Kruskal–Wallis rank sum test for continuous measures. PFS and OS were estimated with the Kaplan–Meier method, stratified by TSG-alt status and treatment, with censoring at the next therapy or last follow-up. The log-rank test was used to assess differences between groups. Cox regression was used for multivariable analysis, using clinical variables associated with worse prognosis such as high disease volume and metastatic disease at diagnosis. R statistical software version 3.6.2 was used for analysis. *P* < 0.05 were considered statistically significant.

## Results

We identified 97 patients with M1-HSPC who met all inclusion criteria during the study period. Patient baseline characteristics are shown in Table 1. The median age was 70 years (range, 41–89 years). A total of 38 patients (39%) had received prior treatment with either prostatectomy or ADT in combination with localized radiation. The analyzed tissue samples were from radical prostatectomies (*n* = 11), prostate biopsies (*n* = 39), transurethral resection of the prostate specimens (*n* = 9), and biopsy of a local or distant metastatic site (*n* = 38). Most patients (66%) had de novo metastatic disease at presentation, and 63 (65%) met high-volume disease criteria. The treatment regimen was ADT + D for 49 patients (51%) and ADT + A for 48 patients (49%).

**Table 1:** Patient characteristics by treatment group^a^.

Characteristic	All patients (*N* = 97)	Treatment		*P* value
		Abiraterone acetate (*n* = 48)	Docetaxel (*n* = 49)	
Prior treatment				0.03^b^
None	59 (61)	23 (48)	36 (73)	
Surgery	28 (29)	19 (40)	9 (18)	
RT	10 (10)	6 (13)	4 (8)	
Mets at diagnosis	64 (66)	27 (56)	37 (76)	0.045^b^
TSG alterations				
* TP53*	35 (36) (*n* = 96)	17 (35)	18 (38) (*n* = 48)	0.83^b^
* PTEN*	30 (31)	17 (35)	13 (27)	0.34^b^
* BRCA*	16 (16)	11 (23)	5 (10)	0.09^b^
* APC*	10 (10)	3 (6)	7 (14)	0.19^b^
* AR*	11 (11)	9 (19)	2 (4)	0.02^b^
* RB1*	6 (6)	3 (6)	3 (6)	0.98^b^
Total TSG alterations	(*n* = 96)		(*n* = 48)	0.93^b^
None	45 (47)	21 (44)	24 (50)	
1	33 (34)	18 (38)	15 (31)	
2	16 (17)	8 (17)	8 (17)	
3	2 (2)	1 (2)	1 (2)	
MDB				0.009^b^
Low	34 (35)	23 (48)	11 (22)	
High	63 (65)	25 (52)	38 (78)	
Visceral progression	29 (30)	15 (31)	14 (29)	0.77^b^
Bone progression	61 (64) (*n* = 96)	29 (62) (*n* = 47)	32 (65)	0.71^b^
Laboratory values				
Alkaline phosphatase, IU/L	103.0 (13.0–1380.0) (*n* = 96)	99.0 (66.0–260.0)	108.5 (13.0–1380.0) (*n* = 48)	0.050^c^
LDH, U/L	191.0 (130.0–407.0) (*n* = 80)	194.0 (131.0–247.0) (*n* = 47)	186.0 (130.0–407.0) (*n* = 33)	0.81^c^
Hemoglobin, g/dL	12.5 (7.4–16.1) (*n* = 95)	12.2 (9.0–15.0) (*n* = 48)	13.0 (7.4–16.1) (*n* = 47)	0.52^c^

*LDH* lactate dehydrogenase, *MDB* metastatic disease burden, *Mets* metastases, *RT* radiotherapy, *TSG* tumor suppressor gene.

^a^Values are No. of patients (%) or median (range).

^b^Pearson χ^2^ test.

^c^Kruskal–Wallis rank sum test.

TSG-alt was present in 51 of 96 patients with data available (53%): *TP53* in 36%, *PTEN* in 31%, and *RB1* in 6% (Table 1). Thirty-three patients (34%) had only 1 TSG-alt, whereas 18 (19%) had more than one TSG-alt: *TP53* + *PTEN* in 14 patients, *PTEN* + *RB1* in two patients, and all three in two patients. Clinical characteristics, prior locoregional treatment, disease volume, baseline laboratory values, and metastatic disease at diagnosis were similar between TSG-alt and TSG-normal groups (Supplementary Table 2).

**Fig. 1:**
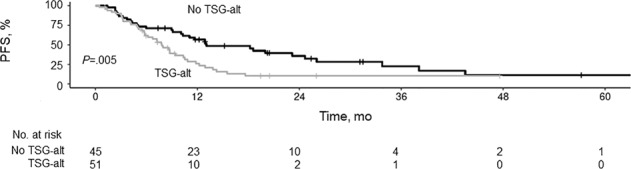
Progression-Free Survival (PFS) by Presence of Tumor Suppressor Gene Alteration (TSG-alt). Kaplan–Meier curves showing PFS for patients with and without TSG-alt.

**Fig. 2:**
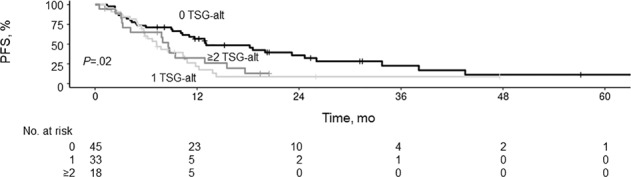
Progression-Free Survival (PFS) by Number of Tumor Suppressor Gene Alterations (TSG-alt). Kaplan–Meier curves showing PFS for patients with 0, 1, or ≥2 TSG-alt.

Median follow-up duration was 23.2 months (range, 1.1–65.2 months). Comparison of patients with and without TSG-alt showed that median PFS was lower for patients with TSG-alt (7.8 months [95% CI, 5.8–10.5 months]) than for TSG-normal patients (13.1 months [95% CI, 10.3–26.0 months]; *P* = 0.005) (Fig. 1). Similarly, median OS differed between the TSG-alt (27.6 months [95% CI, 19.0 months–not estimated]) and TSG-normal group (53.3 months [95% CI, 38.8 months–not estimated]; *P* = 0.03). Patients without TSG-alt had better PFS than those with any TSG (Fig. 2). There was no difference in median PFS, however, between those having only 1 TSG-alt (7.3 months [95% CI, 5.8–10.8 months]) vs. >1 TSG-alt (8.6 months [95% CI, 4.2–17.6 months]).

**Fig. 3:**
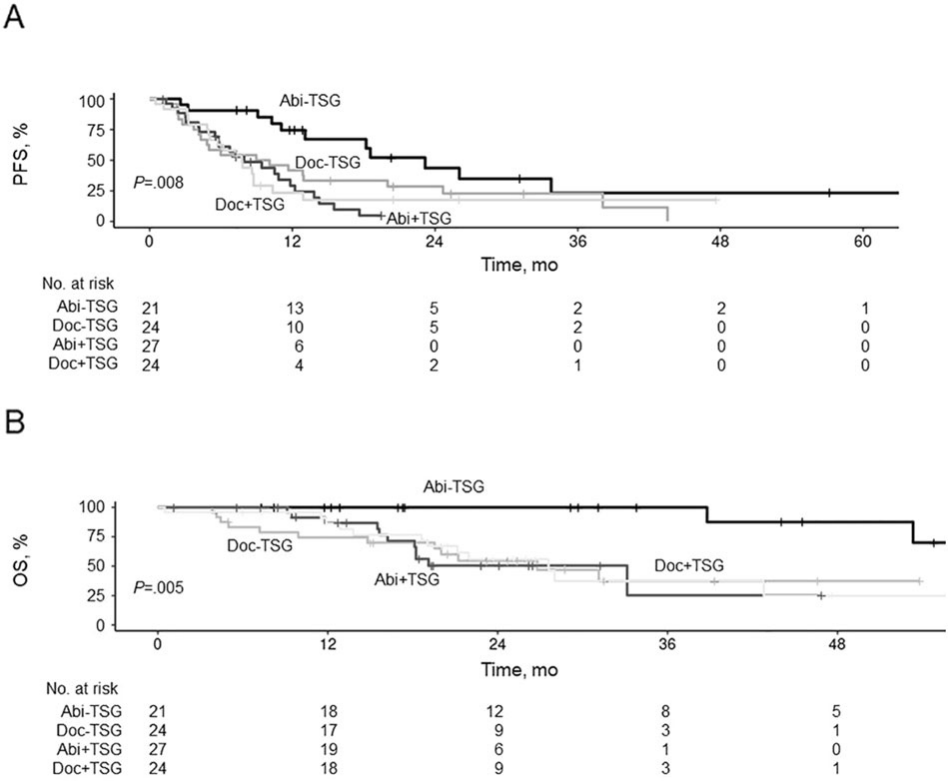
Progression-Free Survival (PFS) and Overall Survival (OS) by First-Line Treatment and Tumor Suppressor Gene Alteration (TSG-alt) Status. Kaplan–Meier curves showing PFS (**A**) and OS (**B**) for patients with (+TSG) and without (−TSG) TSG-alt treated with abiraterone acetate (Abi) or docetaxel (Doc).

There was no substantial difference in median PFS in the ADT + A group (11.8 months [95% CI, 9.4–18.2 months]) vs. the ADT + D group (8.6 months [95% CI, 5.7–12.9 months]) for the total cohort. Within treatment groups, however, median PFS was lower for patients with TSG-alt vs. TSG-normal for those with ADT + A (TSG-alt: 8.0 [95% CI, 5.8–13.8] months vs. TSG-normal: 23.2 [95% CI, 13.1–not estimated] months), but not with ADT + D (TSG-alt: 7.8 [95% CI, 5.7–12.9] months vs. TSG-normal: 9.5 [95% CI, 4.8–24.7] months) (Fig. 3A). Similar results were seen for OS, with ADT + A with TSG-normal being superior to the other groups (Fig. 3B).

On multivariable analysis, TSG-alt and metastatic disease at diagnosis were the only characteristics associated with PFS (Table 2). TSG-alt predicted worse prognosis (hazard ratio, 2.37; 95% CI, 1.42−3.96; *P* < 0.001), whereas metastatic disease predicted improved PFS (hazard ratio, 0.34; 95% CI, 0.15–0.76; *P* = 0.009). The association of TSG-alt with OS did not reach statistical significance on multivariable analysis (hazard ratio, 1.94; 95% CI, 0.98–3.86; *P* = 0.06) (Table 2).

**Table 2:** Multivariable cox regression, PFS and OS.

Characteristic	PFS		OS	
	HR (95% CI)	*P*	HR (95% CI)	*P*
TSG-alt (vs TSG-normal)	2.37 (1.42–3.96)	<0.001	1.94 (0.98–3.86)	0.06
Treatment with ADT + D (vs ADT + A)	1.36 (0.81–2.28)	0.20	1.80 (0.84–3.83)	0.13
Prior treatment (vs none)				
Surgery	1.16 (0.48–2.77)	0.70	0.90 (0.28–2.89)	0.90
RT	0.40 (0.13–1.22)	0.11	0.76 (0.15–3.91)	0.70
High disease volume (vs low)	1.82 (0.99–3.33)	0.05	0.98 (0.41–2.32)	>0.90
Mets at diagnosis (vs none)	0.34 (0.15–0.76)	0.009	1.18 (0.41–3.45)	0.80

*ADT* + *A* androgen deprivation therapy plus abiraterone acetate, *ADT* + *D* androgen deprivation therapy plus docetaxel, *HR* hazard ratio, *Mets* metastases, *OS* overall survival, *PFS* progression-free survival, *RT* radiotherapy, *TSG-alt* tumor suppressor gene alteration.

## Discussion

In the current study, we show that the presence of loss-of-function alterations of *TP53*, *PTEN*, and *RB1* outperforms clinical factors in predicting poor outcomes in M1-HSPC. Using commercially available sequencing assays, we confirmed that the presence of TSG-alt predicts early progression during first-line therapy regardless of treatment selection and has the potential to aid treatment decisions. The presence of TSG-alt also was associated with decreased benefit from abiraterone acetate treatment.

Our findings demonstrated a lower prevalence of *TP53*, *PTEN*, and *RB1* alterations in M1-HSPC than is seen in metastatic, castration-resistant prostate cancer; a previous report in this cohort showed an alteration frequency of 50% for *TP53*, 17% for *PTEN*, and 10% for *RB1* [22]. This is consistent with the evolutionary model of treatment pressure in selecting for resistant clones as disease advances. This is also consistent with aggressive phenotypes of androgen receptor–independent anaplastic and neuroendocrine tumors, characterized by loss of *TP53*, *PTEN*, and *RB1* drivers [23, 24]. Thus, it is not surprising that the early presence of functional TSG losses is prognostic for early progression during first-line treatment with ADT + A or ADT + D.

In our cohort, 18 patients (19%) had evidence of two or more TSG-alt. This rate of TSG coalteration is comparable to the 16% incidence in M1-HSPC, and higher than the 11% incidence in localized hormone-sensitive prostate cancer in the cohorts reported by Hamid et al. [1]. Whether TSG coalteration would confer added prognostic significance or whether loss of a single TSG is enough to confer the full risk of TSG loss will need to be evaluated in larger data sets. Our findings confirm those reported by Stopsack et al. [25] that some genomic alterations were associated with worse prognosis (*AR, MYC*, and *TP53*) and some had better outcomes (*SPOP* and *WNT*). Our study suggests that M1-HSPC with TSG-normal is best treated with ADT + A rather than ADT + D. In the TSG-alt population, outcomes with ADT-D and ADT-A appear similarly poor, which suggests the need for better treatment options for this group.

Genetic characterization in the first-line treatment setting is feasible because all or nearly all patients should have tissue confirmation of their diagnosis. This was recently demonstrated by Gilson and colleagues [26] in their report on genetic analysis in the STAMPEDE study (Systemic Therapy in Advancing or Metastatic Prostate Cancer: Evaluation of Drug Efficacy). The STAMPEDE trial [26] evaluated the feasibility of performing tumor sequencing to design biomarker-directed trials in metastatic castrate-resistant prostate cancer. In two large cohorts of this subset of patients (185 tumors analyzed retrospectively and 101 prospectively), implementing genomic characterization using residual biopsies was shown to be feasible for directing clinical trial designs in the future. Compared with our cohort, that study showed a similar incidence of genomic alterations for *TP53* (33% vs. 36%), *PTEN* (34% vs. 31%), and *RB1* (1% vs. 6%). An advantage of our study is that we were able to compare first-line treatments, instead of comparing randomly assigned therapies in an ongoing clinical trial [26].

Stratification by genetic factors has not yet been included in trials of M1-HSPC. Our current results suggest that TSG status could be a considerable confounding factor for clinical outcomes based on first-line therapy choices. We found a significant difference in PFS and OS between patients with or without TSG-alt who had first-line treatment with abiraterone acetate. In the future, generalized use of broad genomic tumor sequencing in early prostate cancer may be the most accurate method for risk stratification, at least more so than a consensus definition of tumor volume or perhaps even distinguishing de novo vs. relapsed metastatic disease. However, on multivariable analysis, volume of disease and metastatic disease at diagnosis were not particularly associated with PFS and OS.

The main strength of our study is that it includes a real-world patient cohort treated at a tertiary referral center, with sequencing performed using commercially available platforms. This makes our results applicable to most routine oncology practices to identify high-risk patients early in their disease course. In addition, to our knowledge, ours is one of the largest cohorts to date to examine the prognostic significance of TSG status in patients with M1-HSPC [25].

Finally, the different clinical outcomes of our population may result from a slightly higher-risk subset of patients compared with those in historical trials, with regard to known negative prognostic factors such as de novo presentation (61%, vs. 50% in STAMPEDE, 72% in CHAARTED), metastatic disease burden (65%, vs. not reported in STAMPEDE, 64% in CHAARTED), and higher presence of visceral metastases (30%, vs. 6% in STAMPEDE, 15% in CHAARTED) [27]. These differences reflect the variability of highly selected cohorts included in randomized clinical trials in contrast to a heterogeneous real-world patient population.

### Limitations

Our study has several limitations. Because this was a retrospective study, significant confounding factors and selection bias cannot be excluded. To limit selection bias, we included all consecutive patients who met inclusion criteria. However, patients without tumor sequencing were excluded, which limits the generalizability of our results. The modest sample size constrains the statistical significance of our findings, and, thus, the data can only be seen as hypothesis generating. Our study reflects some institutional practice patterns. Another limitation is the suboptimal detection and heterogeneity of comprehensive genomic analyses in some of the gene alterations, particularly *RB1* and *PTEN* [28, 29]. We did not compare genomic results with immunohistochemistry. In contrast to the STAMPEDE [26] and CHAARTED [10] studies, which included criteria for PSA progression, we were unable to retroactively apply PSA progression criteria to patients who continued on treatments as directed by their physician.

## Conclusions

In summary, we confirmed that pathogenic TSG-alt in *TP53*, *PTEN*, and *RB1* in patients with M1-HSPC portend a poor prognosis early in the disease course. Loss of function of these TSGs is prognostic for early progression during first-line treatment, regardless of the treatment selection, and identification of TSG-alt outperforms most clinical variables as a predictor of early progression in multivariable analysis. Increased use of tumor sequencing to detect these alterations early in the presentation of prostate cancer may establish the need for inclusion of molecular stratification in prognostic algorithms. Confirmation of these findings holds the potential for improving prognostic tools to better define high-risk patients.

## Supplementary information

Supplementary Tables
