# Supplementary material for: Differential impact of tumor suppressor gene (TP53, PTEN, RB1) alterations and treatment outcomes in metastatic, hormone-sensitive prostate cancer
Source: Prostate Cancer Prostatic Dis. 2021 Jul 22;25(3):479–83. doi: 10.1038/s41391-021-00430-4 (PMC9385473; doi:10.1038/s41391-021-00430-4)
Supplement: Supplementary file 1 — Supplementary Tables [file 41391_2021_430_MOESM1_ESM.docx]

**Supplemental Table 1.** Frequency of alterations of *TP53*, *PTEN* and *RB1* in all cohorts

|  |  | M1-HSPC  (N=98) |  |
| --- | --- | --- | --- |
| Alteration | | | |
| *TP53* alteration |  | 35 (36%) | |
| *PTEN* alteration |  | 30 (31%) | |
| *RB1* alteration |  | 6 (6%) | |
|  | | | |
| *TP53: Frameshift*  *TP53*: Deletion |  | 27  3 |  |
| *PTEN: Frameshift*  *PTEN*: Deletion |  | 5  25 |  |
| *RB1*: Frameshift |  | 4 |  |
| *RB1*: Deletion |  | 2 |  |

**Supplemental Table 2.** Patient demographics by tumor suppressor genes^a^

|  | TSG-normal (N=45) | TSG-altered (N=51) | Total (N=96) | P value |
| --- | --- | --- | --- | --- |
| **Prior treatment** |  |  |  | 0.741^b^ |
| No | 29 (64.4%) | 29 (56.9%) | 58 (60.4%) |  |
| Surgery | 12 (26.7%) | 16 (31.4%) | 28 (29.2%) |  |
| RT | 4 (8.9%) | 6 (11.8%) | 10 (10.4%) |  |
| **Mets on diagnosis** |  |  |  | 0.819^b^ |
| No | 16 (35.6%) | 17 (33.3%) | 33 (34.4%) |  |
| Yes | 29 (64.4%) | 34 (66.7%) | 63 (65.6%) |  |
| **Treatment** |  |  |  | 0.539^b^ |
| Abiraterone | 21 (46.7%) | 27 (52.9%) | 48 (50.0%) |  |
| Docetaxel | 24 (53.3%) | 24 (47.1%) | 48 (50.0%) |  |
| **MDB** |  |  |  | 0.979^b^ |
| Low | 16 (35.6%) | 18 (35.3%) | 34 (35.4%) |  |
| High | 29 (64.4%) | 33 (64.7%) | 62 (64.6%) |  |
| **Visceral progression** |  |  |  | 0.041^b^ |
| No | 36 (80.0%) | 31 (60.8%) | 67 (69.8%) |  |
| Yes | 9 (20.0%) | 20 (39.2%) | 29 (30.2%) |  |
| **Bone progression** |  |  |  | 0.964^b^ |
| No. missing | 0 | 1 | 1 |  |
| No | 16 (35.6%) | 18 (36.0%) | 34 (35.8%) |  |
| Yes | 29 (64.4%) | 32 (64.0%) | 61 (64.2%) |  |
| **Alkaline phosphatase, IU/L** |  |  |  | 0.917^c^ |
|  | 103.0 (57.0-1,380)  (n=45) | 104.0 (13.0-643.0)  (n=50) | 103.0 (13.0-1,380)  (n=95) |  |
| **LDH, U/L** |  |  |  | 0.389^c^ |
|  | 195.5 (130.0-407.0)  (n=36) | 188.5 (132.0-255.0)  (n=44) | 191.0 (130.0-407.0)  (n=80) |  |
| **Hemoglobin, g/dL** |  |  |  | 0.306^c^ |
|  | 12.0 (9.0-16.0)  (n=43) | 13.0 (7.4-16.1)  (n=51) | 12.4 (7.4-16.1)  (n=94) |  |

Abbreviations: LDH, lactate dehydrogenase; MDB, metastatic disease burden; Mets, metastases; RT, radiotherapy; NGS, next generation ds tumor suppressor gene.

^a^ Values are No. of patients (%) or median (range)

^b^ Pearson χ^2^ test.

^c^ Kruskal-Wallis rank sum test.
